# Growth of a *Saccharomyces cerevisiae *strain lacking hexose transporters in different sugars after transformation with a *Scheffersomyces stipitis *genomic library

**DOI:** 10.1186/1753-6561-8-S4-P122

**Published:** 2014-10-01

**Authors:** Belisa Sales, Davi Gonçalves, Bruna Scheid, Boris Stambuk

**Affiliations:** 1 Universidade Federal de Santa Catarina, Florianópolis, Brazil

## Background

Brazil is the biggest ethanol producer in the world using sugarcane as substrate. From this process, bagasse is the main resulting by-product which can be used to produce second generation ethanol. Although *Saccharomyces cerevisiae *is the ideal yeast for the fermentative process, it cannot ferment xylose, one of the most abundant sugars in lignocellulosic biomass. Different yeast can ferment xylose, including *S. stipitis *[1], but not with the same efficiently rate as *S. cerevisiae*. Genes from xylose-fermenting yeasts can be used to genetically engineer *S. cerevisiae *to improve bioethanol production. Since it is widely known that the transport of sugars inside the cells is a limiting factor for the fermentation of sugars, and several reports have demonstrate that an increase in transport activity also increases the fermentative capacity of yeast cells [2], in the present work we have used a *S. cerevisiae *strain lacking hexose transporters (*hxt*-null) to screen a *S. stipitis *genomic library to identify putative xylose transporter genes.

## Methods

The genomic library was constructed using DNA from *S. stipitis *by a private company (requested by Dr. Matsuhika from AIST, Japan) and the fragmented DNA was cloned into the overexpressing plasmid pPGK. These plasmids were used to transform a *S. cerevisiaehxt*-null strain (which can grow only in maltose) previously transformed with the integrative plasmid pAUR-XKXDHXR [3] that confers the cells the capacity to metabolize xylose. The clones were first select in solid synthetic medium with 2% xylose. Growth in micro-scale using synthetic medium with 2% maltose, xylose, glucose, fructose, galactose or mannose as carbon sources was performed using a Tecan Microplate reader (Tecan Echisto Infinite M200PRO) for up to 72 h at 28°C under orbital agitation (160 rpm). The absorbance was measured at 570nm every 15 minutes.

## Results and conclusions

Genomic libraries from different yeast on multi-copy plasmids can reveal potential genes [4] for the improvement of xylose transport in *S. cerevisisae*. We obtained 90 *S. cerevisiae *transformants from the *S. stipitis *genomic library, and 8 clones were selected based on their capacity to grow on xylose and glucose synthetic medium, although the final absorbance in xylose was lower than when glucose was the carbon source (data not shown). Two transformants, BBY-D1Ss6 and BBY-D1Ss90, grow higher than the other transformants in all the sugars tested, with a similar and marked growth in mannose. In contrast, BBY-D1Ss41 and BBY-D1Ss53 transformat strains did not grow on this sugar. The growth of strain BBY-D1Ss80 was also expressive in all sugars (mostly in glucose and fructose). In general, the eight transformants were able to grow on different sugars, not been specific to one carbohydrate neither specific for glucose only. We are analyzing the inserts present in the pPGK plasmid to verify which genes from the *S. stipitis *genome have been isolated in our work.
